# Adaptive Changes in Glucose Homeostasis and Islet Function During Pregnancy: A Targeted Metabolomics Study in Mice

**DOI:** 10.3389/fendo.2022.852149

**Published:** 2022-05-04

**Authors:** Ziyi Zhang, Anthony L. Piro, Feihan F. Dai, Michael B. Wheeler

**Affiliations:** ^1^ Department of Physiology, Faculty of Medicine, University of Toronto, Toronto, ON, Canada; ^2^ Department of Endocrinology, Sir Run Run Shaw Hospital, Zhejiang University, Hangzhou, China; ^3^ Metabolism Research Group, Division of Advanced Diagnostics, Toronto General Hospital Research Institute, Toronto, ON, Canada

**Keywords:** pregnancy, metabolic adaptions, mouse, islet metabolism, targeted metabolomics

## Abstract

**Objective:**

Pregnancy is a dynamic state involving multiple metabolic adaptions in various tissues including the endocrine pancreas. However, a detailed characterization of the maternal islet metabolome in relation to islet function and the ambient circulating metabolome during pregnancy has not been established.

**Methods:**

A timed-pregnancy mouse model was studied, and age-matched non-pregnant mice were used as controls. Targeted metabolomics was applied to fasting plasma and purified islets during each trimester of pregnancy. Glucose homeostasis and islet function was assessed. Bioinformatic analyses were performed to reveal the metabolic adaptive changes in plasma and islets, and to identify key metabolic pathways associated with pregnancy.

**Results:**

Fasting glucose and insulin were found to be significantly lower in pregnant mice compared to non-pregnant controls, throughout the gestational period. Additionally, pregnant mice had superior glucose excursions and greater insulin response to an oral glucose tolerance test. Interestingly, both alpha and beta cell proliferation were significantly enhanced in early to mid-pregnancy, leading to significantly increased islet size seen in mid to late gestation. When comparing the plasma metabolome of pregnant and non-pregnant mice, phospholipid and fatty acid metabolism pathways were found to be upregulated throughout pregnancy, whereas amino acid metabolism initially decreased in early through mid pregnancy, but then increased in late pregnancy. Conversely, in islets, amino acid metabolism was consistently enriched throughout pregnancy, with glycerophospholid and fatty acid metabolism was only upregulated in late pregnancy. Specific amino acids (glutamate, valine) and lipids (acyl-alkyl-PC, diacyl-PC, and sphingomyelin) were found to be significantly differentially expressed in islets of the pregnant mice compared to controls, which was possibly linked to enhanced insulin secretion and islet proliferation.

**Conclusion:**

Beta cell proliferation and function are elevated during pregnancy, and this is coupled to the enrichment of islet metabolites and metabolic pathways primarily associated with amino acid and glycerophospholipid metabolism. This study provides insight into metabolic adaptive changes in glucose homeostasis and islet function seen during pregnancy, which will provide a molecular rationale to further explore the regulation of maternal metabolism to avoid the onset of pregnancy disorders, including gestational diabetes.

## Introduction

Pregnancy is a dynamic and complex state requiring physiological adaptations that ensure a continuous supply of essential nutrients to support the development and growth of the fetus. These adaptions also prepare mothers for the postnatal support period, and most importantly, to facilitate lactation. Understanding maternal metabolism during gestation is important not only for understanding what promotes a healthy environment for fetal development, but also for identifying and understanding metabolic disorders associated with pregnancy, including gestational diabetes mellitus (GDM) and future risk of type 2 diabetes (T2D).

Glucose metabolism is a key component of the metabolic response to pregnancy gradually changing over the course of gestation to meet the demands of the fetus. Fasting glucose was observed to be significantly decreased in pregnant women, with a concomitant increase in fasting insulin (1); whereas insulin sensitivity was found to be unchanged or increased during early pregnancy, but significantly impaired in later pregnancy (2, 3). Pregnancy is also characterized by altered maternal lipid metabolism, including an anabolic phase at early pregnancy and a catabolic phase at later pregnancy (4). Besides metabolic adaptions in the circulation, pancreatic islets also undergo major structural and functional adaptions in response to the enhanced demand for insulin, including: 1) increased glucose-stimulated insulin secretion, 2) lower threshold for glucose-stimulated insulin secretion, 3) increased insulin biosynthesis, 4) increased proliferation, and 5) increased glucose metabolism (5–9).

Targeted metabolomics is a quantitative assay platformed to assess specific metabolites associated with known biochemical pathways or disease processes. Metabolomic profiling has been instrumental in developing metabolic databases and identifying potential disease biomarkers or metabolic pathways associated with diseases like GDM and T2D associated with a GDM history (10–13). Our previous studies have demonstrated the onset of future T2D after GDM pregnancy was associated with upregulation of glycolipid metabolism involving triacylglycerol and diacylglycerol biosynthesis; but decreased sphingolipid/phospholipid metabolism (11, 12, 14). The inhibition of sphingolipid metabolism in islets led to impaired pancreatic beta cell function (14, 15). However, studies applying metabolomics to evaluate metabolic changes associated with pregnancy are limited, particularly those combined with measurements of specific metabolic parameters including islet and beta cell function. Several studies have applied metabolomics to define biomarkers of GDM (16–21), but have yielded inconsistent results, potentially due to a host of contributory factors (i.e. small sample sizes, different methodology, various treatments, etc.). At present, no studies have tracked longitudinally changes in the circulating and pancreatic islet metabolome over the course of a normal pregnancy. Such studies would reveal alterations in the metabolome in circulation and islets that coincide with adaptations in islet growth and function over the course of pregnancy.

In this present preclinical investigation, a timed-pregnancy mouse model was used to evaluate maternal metabolic adaptive changes in circulation and pancreatic islets at different phases of a normal pregnancy. Changes in the metabolome were then used to identify specific metabolites and metabolic pathways associated with adaptations in islet function during the trimesters of pregnancy.

## Materials and Methods

### Animals

FVB female and male mice of 8 weeks old were used for timed-pregnancy mouse model generation. FVB female and male mice were paired overnight in 1:2 (male: female) pairs. The next morning, the presence of a copulatory plug was checked by trained staff to confirm the successful mating. Age-matched non-pregnant FVB female mice were used as controls. All female mice have never been pregnant prior to this study. Mice with gestational day (GD) 0.5-14 correspond to the first trimester in humans, during which preimplantation and post-implantation events occur. Mice with GD 14-17 are compared to the second trimester in humans, which encompasses fetal and placental growth. Mice with GD 17-birth align with the third trimester in humans when fetal growth is accelerated. These time points are highly translatable to humans and represent the three trimesters during human pregnancy (22). A total of 5-7 pregnant or age-matched non-pregnant mice were used in each trimester to evaluate glucose homeostasis, islet function and islet proliferation. Three groups of 500 islets isolated and pooled from 2-3 pregnant or non-pregnant mice in each trimester were subjected to targeted metabolomics analysis. Targeted metabolomics was also performed on fasting plasma samples collected from 4 pregnant or non-pregnant mice in each trimester. All animal experiments and methods have been approved by the University of Toronto animal care committee (#20011576).

### Oral Glucose Tolerance Test (OGTT) and Plasma Insulin Measurement

Mice were fasted overnight (14-16 hours), and a 2 g/kg bolus of glucose was administered by oral gavage as previously described (23, 24). Briefly, blood glucose was measured using a Contour glucometer (Ascensia Diabetes Care, Mississauga, ON, Canada) at 0, 10, 30, 60, 90 and 120 mins. Blood samples were also collected from tail vein at 0, 10 and 30 mins for plasma insulin measurements. Fed glucose and insulin samples were collected at 9 am in each trimester. Plasma was separated by centrifugating at 6,000 rpm for 10 mins at 4°C. Insulin levels were then analyzed by using a mouse supersensitive insulin ELISA (ALPCO, Salem, NH, USA) (25, 26).

### Mouse Islets Isolation

Pregnant and age-matched non-pregnant mouse islets were isolated as described previously (27, 28). Briefly, mice were anesthetized *via* isoflurane and cervical dislocation was performed. The ampulla was clamped with surgical suture to block the bile pathway to the duodenum. Collagenase V solution was prepared at 0.8 mg/mL using 1640 RPMI (Sigma-Aldrich, Burlington, MA, USA). A needle was inserted in the bile duct and 3 mL of prepared Collagenase V solution was slowly dispersed. The well-perfused pancreas was removed and placed in 5 mL of prepared Collagenase V solution. Pancreas were then digested at 37.0°C for 11 mins followed by a brief shake to produce a homogeneous digestive solution. The digestion was terminated by adding 50 mL of full medium (1640 RPMI + 10% FBS + 1% penicillin/streptomycin). Islets were hand-picked three times and allowed to recover overnight in full medium prior to further experiments.

### Glucose Stimulated Insulin Secretion

Glucose-stimulated insulin secretion studies were carried out as previously described using 2.0 mM as low glucose and 11.0 mM as high glucose (29, 30). Secreted insulin concentrations were quantified by Homogenous Time-Resolved Fluorescence Kit (Cisbio, Codolet, France) and the results were normalized to total DNA content.

### Immunohistochemistry

The whole pancreas was dissected from each mouse and was fixed in 10% neutral buffered formalin for 4-24 hour before being embedded in paraffin. The whole pancreas was stretched in the block to maximize the total pancreatic area. Sections close to the middle layer which represents the largest pancreatic area were used for insulin, glucagon and Ki67 staining as previously described (27, 28). Images were obtained using the Zeiss Axioscan Slide Scanner and all image quantifications were carried out by HALO (Indica Labs, v.2.0.1145.14; Corrales, NM, USA). Beta-cell and alpha-cell mass was calculated by multiplying the average insulin- or glucagon-positive area in relation to the whole pancreatic area with the pancreatic weight of corresponding animal, as previously described (31). The counted beta-cell number on each section was normalized to the corresponding whole pancreatic area of the section. Individual beta-cell size was calculated by dividing the insulin-positive area by the beta-cell number of a given section. Islet size distribution was calculated by obtaining the percentage of islet numbers in designated size ranges.

### Immunofluorescence and Confocal Microscopy

Immunofluorescence staining was performed as previously described (27, 28). Briefly, intact islets were dispersed into single islet cells using TrypLE (Thermo Fisher Scientific, Waltham, MA, USA). Dispersed islets were then loaded onto the slides using a Shandon Single Cytofunnel (Thermo Fisher Scientific). The slides were fixed with 4% paraformaldehyde and incubated with primary anti-insulin (Agilent Technologies, Santa Clara, CA, USA), anti-glucagon (Abcam, Cambridge, MA, USA), and anti-Ki67 (Abcam) overnight at 4°C. Secondary anti-guinea pig Alexa488 (Thermo Fisher Scientific), anti-mouse Alexa555 (Abcam) and anti-rabbit Cy5 (Thermo Fisher Scientific) were used to detect the target proteins. Images were obtained using a Zeiss Axioscan Slide Scanner (Zen, Blue Edition, v.2.3.69.1000; Carl Zeiss GmbH). All image quantifications were carried out by HALO (Indica Labs, v.2.0.1145.14; Corrales, NM, USA). Cell proliferation rate was calculated by normalizing Ki67+ insulin+/glucagon+ cells to total insulin+/glucagon+ cells.

### Targeted Metabolomics and Data Pre-Processing

The metabolomics analyses were carried out as described previously (12, 13). A total of 500 islets were isolated and collected from the pregnant or non-pregnant group in each trimester. Targeted metabolomics was performed on fasting plasma samples and isolated islets. In this study, the AbsoluteIDQ p180 kit (Biocrates Life Sciences, Innsbruck, Austria) was used to quantify the metabolites and explore the diverse physiological processes. This platform allows the detection of up to 188 metabolites by using mass-spectrometry-based techniques, including hexose, amino acids (AAs), acylcarnitines (ACs), biogenic amines (BAs), glycerophospholipids, and sphingomyelins (SMs). All analyses were performed by the Analytical Facility for Bioactive Molecules (The Hospital for Sick Children, Toronto, ON, Canada) without disclosure of group allocation. For the data pre-processing, metabolites with missing values >40% were excluded from the study, which reduced the total number of metabolites from 188 to 139 in plasma samples and from 188 to 103 in islet samples. The remaining missing values were imputed with half of the limit of detection (LOD) value of each metabolite. The value of each metabolite was normalized within the total value of each sample, followed by log-transformation and mean-centric scaling; distribution of data was then checked. The data pre-processing was performed on the online platform MetaboAnalyst 5.0 (https://www.metaboanalyst.ca/home.xhtml) (32). To further identify the differentially expressed metabolites between pregnant and non-pregnant mice, independent two-tailed student’s t-test was carried out to evaluate significance. Afterwards, false discovery rate (FDR) was calculated using Benjamini-Hochberg method for multiple comparison. Given the limited sample numbers, metabolites with FDR value<0.3 were considered to be significantly differentially expressed between pregnant and non-pregnant mice.

### Fuzzy C-Means Clustering and KEGG Pathway Analysis

Fuzzy C-means clustering analysis was performed to identify the clusters of metabolites with similar dynamic trends during the three phases of pregnancy (33) in RStudio (Version 1.2.5033) using the package “Mfuzz”. The minimum centroid distance between clusters for a series of cluster numbers was calculated to determine the appropriate cluster number in plasma and islet samples. Then metabolites within each cluster were subjected to pathway analysis using the Kyoto Encyclopedia of Genes and Genomes (KEGG, Kanehisa Laboratories, Kyoto, Japan) database. The KEGG pathway analysis was performed on MetaboAnalyst 5.0.

### Statistics

The Shapiro–Wilk normality test was used to determine data normality. Mann–Whitney U, unpaired student's t-test, and one- or two-way ANOVA were applied to determine statistical significance where applicable. P-values of less than 0.05 were regarded as statistically significant.

## Results

### Study Workflow

**Figure 1:**
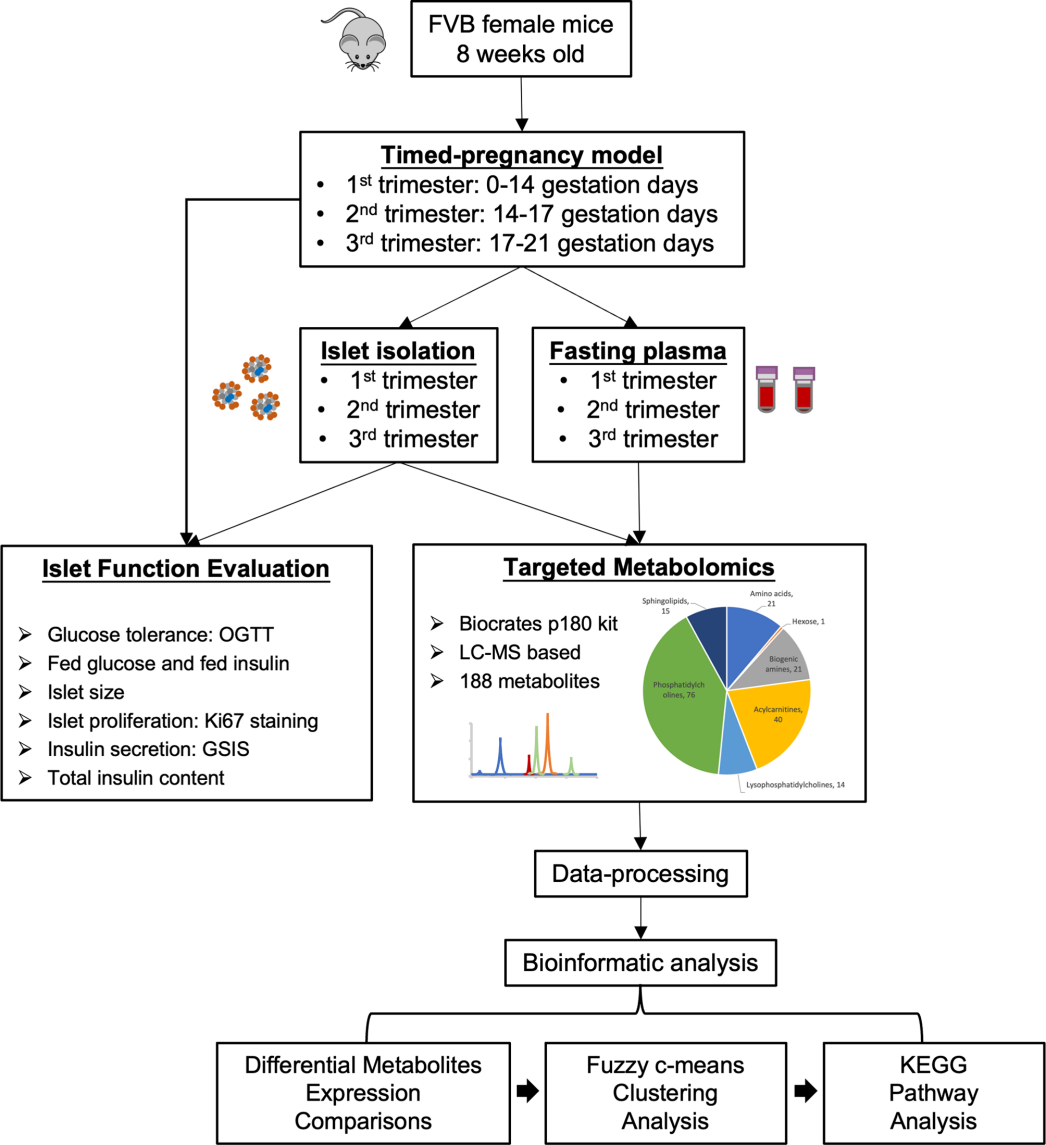
Study design and workflow of the present study. A timed pregnancy mouse model was generated using FVB female mice (1st trimester, 0-14 gestation days; 2nd trimester, 14-17 gestation days; 3rd trimester, 17-21 gestation days) and age-matched non-pregnant mice were used as controls. Purified islets and fasting plasma samples were collected during each trimester. Islet function and proliferation were assessed. Islets and fasting plasma samples collected at each trimester were subjected to targeted metabolomics to identify the metabolic profiles in circulation and islets. Bioinformatics analyses (differential expression analysis, Fuzzy c-means clustering and KEGG pathway analysis) were then performed to evaluate dynamic changes in islet and maternal metabolism throughout the pregnancy.

In the present study we aimed to evaluate changes in plasma and islet metabolomes in response to normal pregnancy in a mouse model by applying targeted metabolomics. **Figure 1** outlines the study design and workflow. Timed-pregnancy mouse models studied at three time-points representing each of the three trimesters were generated using FVB female mice starting at 8 weeks of age. An OGTT was carried out to evaluate glucose homeostasis during each trimester. Fed glucose and fed insulin were also measured in each trimester. Pregnant and non-pregnant mice at each of the three trimesters were sacrificed and islets isolated and collected. Islet size, islet proliferation rate, glucose-stimulated insulin secretion and total insulin content were evaluated. Fasting plasma and purified islet tissue were subjected to a targeted metabolomics screen that included a wide scope of metabolite classes, including over 50 metabolites identified as associated with diabetes and prediabetes pathology (10, 12). Comparisons of metabolites in circulation and islets between pregnant and non-pregnant mice in each trimester were summarized. A Fuzzy c-means clustering was then applied to identify the clusters of metabolites whose levels followed a similar trend throughout the pregnancy. Metabolites within the clusters were then subjected to KEGG pathway analysis to further illustrate the metabolic pathway changes during pregnancy both in circulation and islets.

### Whole Body Metabolism and Glucose Homeostasis Changes During Pregnancy

**Figure 2:**
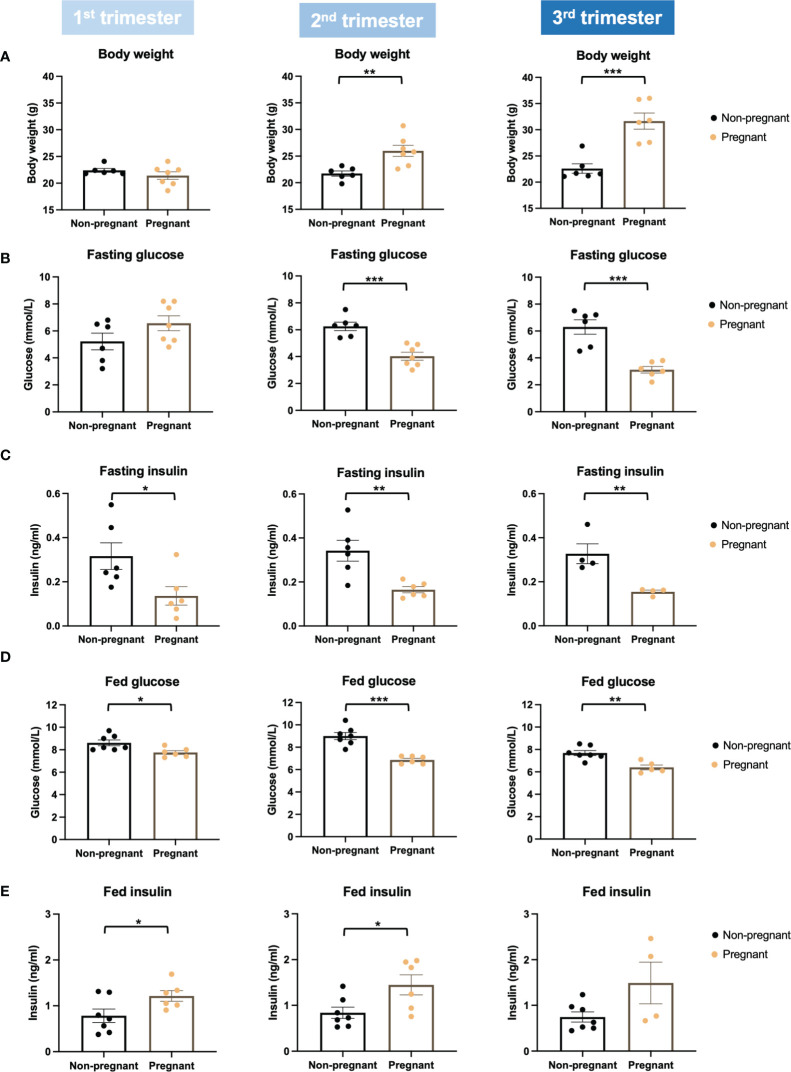
Body weight, glucose, and insulin levels in pregnant and non-pregnant mice at the same timepoints within the three trimesters. **(A)** Body weight. **(B)** Fasting glucose levels. **(C)** Fasting insulin. **(D)** Fed glucose. **(E)** Fed insulin. *P < 0.05, **P < 0.01, ***P < 0.001, compared to age-matched non-pregnant FVB female mice.

**Figure 3:**
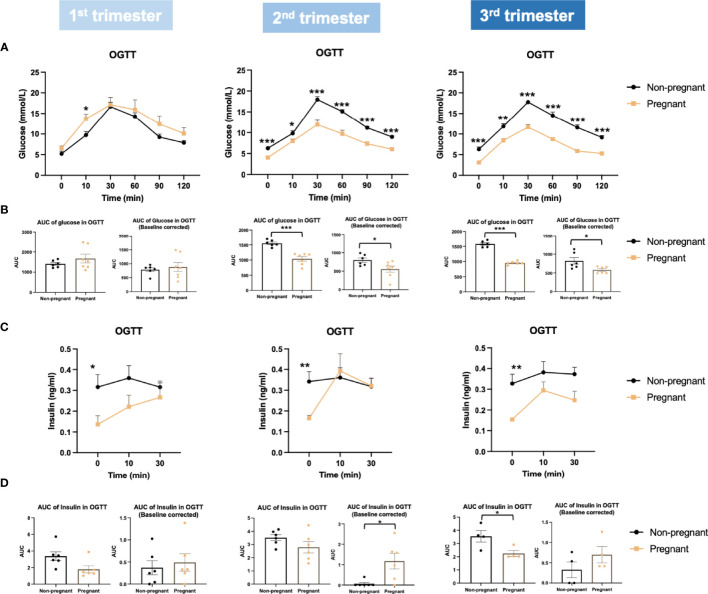
Glucose and insulin levels during oral glucose tolerance test (OGTT) in the three trimesters of pregnancy. **(A)** Glucose levels during OGTT. **(B)** Baseline corrected and baseline non-corrected area under the curve (AUC) of glucose during OGTT. **(C)** Insulin secretion during OGTT. **(D)** Baseline corrected and baseline non-corrected AUC of insulin during the first 10 minutes of OGTT. *P < 0.05, **P < 0.01, ***P < 0.001, compared to age-matched non-pregnant FVB female mice.

Firstly, we evaluated glucose metabolism and insulin response during normal pregnancy. Bodyweight was increased significantly in the 2^nd^ and 3^rd^ trimesters in pregnant mice (2^nd^ trimester, p = 0.005; 3^rd^ trimester, p = 0.0005) (**Figure 2A**). Compared to non-pregnant mice, fasting glucose of pregnant mice began to decrease significantly in the 2^nd^ trimester and continued into the 3^rd^ trimester (2^nd^ trimester, p = 0.0003; 3^rd^ trimester, p = 0.0003) (**Figure 2B**). Fasting insulin was also significantly lower in pregnant mice throughout pregnancy (1^st^ trimester, p = 0.03; 2^nd^ trimester, p = 0.005; 3^rd^ trimester, p = 0.009) (**Figure 2C**). Similar to fasting, fed glucose levels were significantly lower throughout pregnancy (1^st^ trimester, p = 0.02; 2^nd^ trimester, p = 0.0001; 3^rd^ trimester, p = 0.003); however, fed insulin levels in pregnant mice were higher than non-pregnant mice (1^st^ trimester, p = 0.046; 2^nd^ trimester, p = 0.028; 3^rd^ trimester, p = 0.070) (**Figures 2D, E**). We then performed an OGTT to evaluate glucose disposal and insulin response after a glucose load. When correcting to the basal glucose level, the area under the curve (AUC) of glucose was significantly lower in pregnant mice in the 2^nd^ and 3^rd^ trimesters (2^nd^ trimester, p = 0.04; 3^rd^ trimester, p = 0.03), suggesting a higher glucose disposal during pregnancy (**Figures 3A, B**). After correcting for the baseline insulin level, the AUC of insulin secretion during the first 10 minutes of OGTT were higher in pregnant mice, especially in the 2^nd^ trimester (p = 0.03), suggesting a more robust insulin response after a glucose load in pregnant mice compared to non-pregnant ones (**Figures 3C, D**).

### Dynamic Changes of Circulating Metabolites and Pathways During Normal Pregnancy

**Figure 4:**
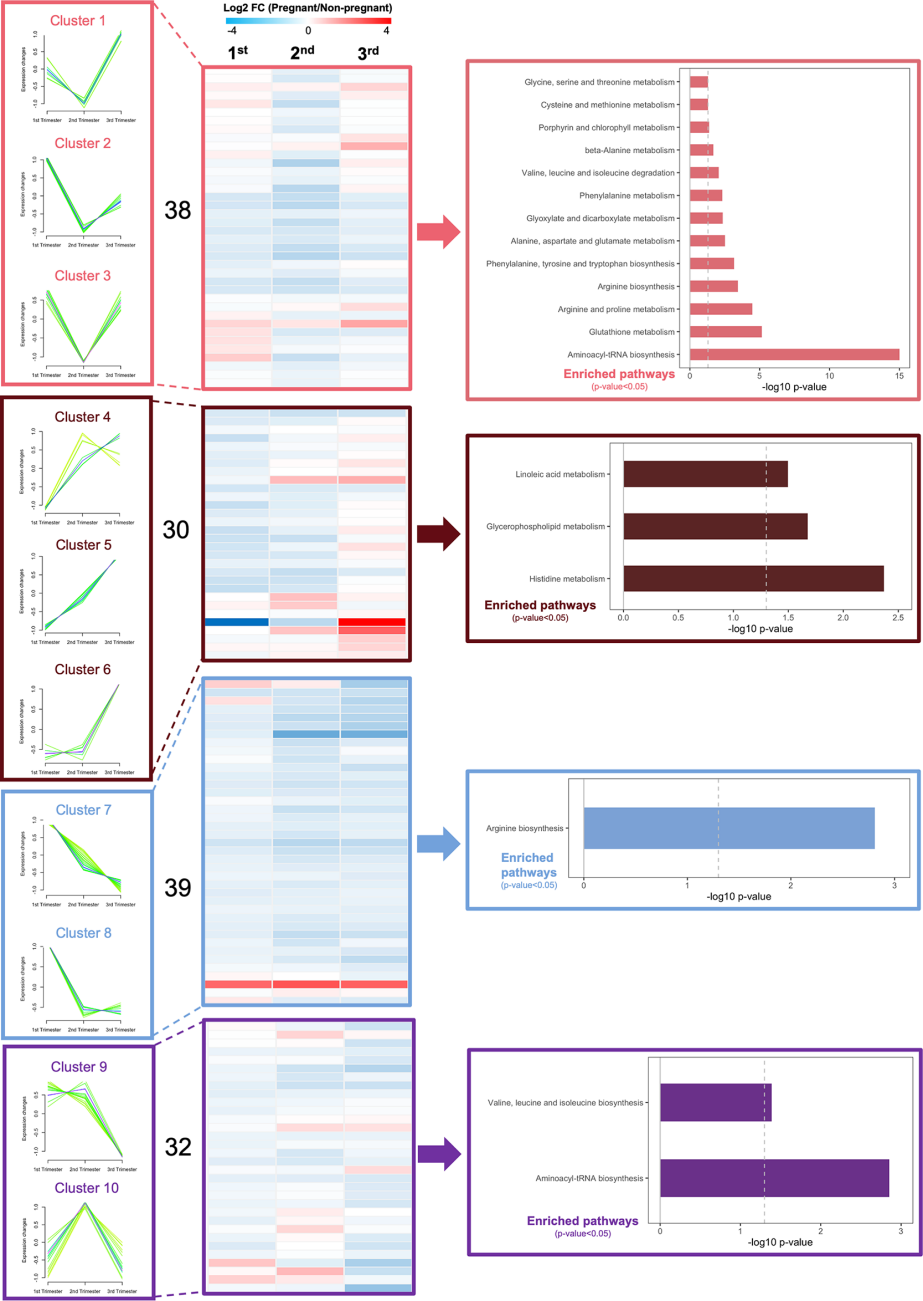
Dynamic changes in metabolic profiles of fasting plasma during the three trimesters of pregnancy. Fuzzy c-means soft clustering was applied to identify the clusters of metabolites with the same trends during pregnancy. KEGG pathway analysis was performed to identify the major regulating signaling pathways within the clusters with the same trend. Fold changes are log transformed and indicated by color scale in the matrix. In heatmaps, red color indicates up-regulated in pregnant mice, whereas blue represents down-regulated in pregnant mice, compared to non-pregnant and age-matched mice. A total of 4 pregnant and non-pregnant mice were used in each trimester.

To better understand metabolic changes that occur during pregnancy, we evaluated circulating metabolites over the three trimesters, by applying targeted metabolomics to fasting plasma samples. We identified 139 metabolites in circulation, including 21 amino acids (AAs), 10 acylcarnitines (ACs), 15 biogenic amines (BAs), 1 monosaccharide, 78 glycerophospholipids, and 14 sphingomyelins (SMs). By comparing pregnant to non-pregnant mice, the log2 fold change value of each metabolite was calculated and shown in heatmaps (**Supplementary Figure 1**). Using fuzzy c-means clustering, we then identified 10 major clusters of metabolites (**Figure 4**). The top significantly regulated pathways of each cluster of metabolites were identified using KEGG pathway analysis (p-value<0.05) (**Figure 4**). Assimilating this data, comparing pregnant mice to non-pregnant mice, AA metabolism pathways were downregulated from 1^st^ to 2^nd^ trimester and upregulated in very late pregnancy (3^rd^ trimester), close to birth. The AA metabolic pathways identified included glutathione metabolism, arginine and proline metabolism, phenylalanine, tyrosine and tryptophan biosynthesis, etc. (**Figure 4** and **Supplementary Figure 1**, clusters 1-3). In addition, glycerophospholipid and fatty acid metabolism pathways were steadily upregulated throughout the three phases of pregnancy (**Figures 4** and **Supplementary Figure 1**, clusters 4-6).

Additionally, we identified significantly differentially expressed metabolites between pregnant and non-pregnant mice within each trimester (**Supplementary Figure 2**). In the 1^st^ trimester, 79 out of 139 metabolites detected were found to be significantly differentially expressed (FDR<0.3), including 11 sphingomyelins, 59 phospholipids, 4 amino acids, 3 acylcarnitines, 1 biogenic amine and hexose. Most of these analytes were downregulated in pregnant mice (**Supplementary Figure 2**). In the 2^nd^ trimester, 5 sphingomyelins, 9 phospholipids, 3 biogenic amines, 2 acylcarnitines and 4 amino acids were significantly changed. Most lipid species (except lyso PCs) and amino acids were upregulated in pregnant mice. In the 3^rd^ trimester, 16 phospholipids, 5 biogenic amines, 8 amino acids and hexose were significantly differentially expressed. The majority of these metabolites (except lyso PCs, valine, tyrosine, and hexose) were significantly higher in pregnant mice. Overall, in circulation, most differentially expressed analytes were clustered in lipids, including phospholipids and sphingomyelins. In contrast to the changes of lipids, amino acids and biogenic amines were marginally changed between pregnant and non-pregnant mice, suggesting amino acid metabolism remains more stable, while the lipids are largely utilized for pregnancy and nutrient supply for infants.

### Islet Proliferation and Islet Function During Normal Pregnancy

**Figure 5:**
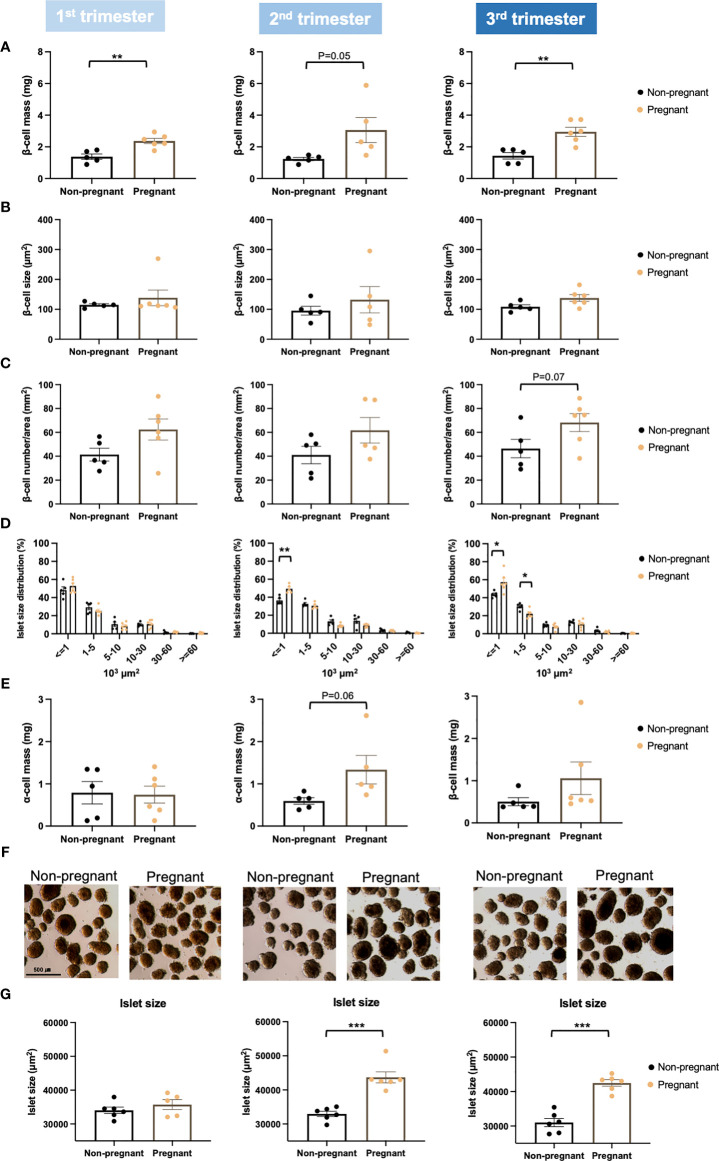
Islet proliferation in pregnant and non-pregnant mice during the three trimesters of pregnancy. **(A)** Beta-cell mass. **(B)** Individual beta-cell size. **(C)** Beta-cell number/area. **(D)** Islet size distribution. **(E)** Alpha-cell mass. **(F, G)** Isolated islet size. Beta-cell mass was determined by multiplying the pancreas weight by the percentage of insulin-positive area in relation to whole pancreas section area. Beta-cell size was calculated by dividing the insulin-stained area by the total beta-cell number. Beta-cell number was counted with HALO (version 2.0.1145.14) and was normalized by whole tissue area. A total of 5-7 pregnant and non-pregnant mice were used in each trimester. *P < 0.05, **P < 0.01, ***P < 0.001 compared to age-matched, non-pregnant FVB female mice.

To assess islet adaptions during pregnancy, islet proliferation and islet function were evaluated. Pregnant mice showed significantly increased beta-cell mass throughout the pregnancy (1^st^ trimester, p = 0.003; 2^nd^ trimester, p = 0.05; 3^rd^ trimester, p = 0.002) (**Figure 5A**). Importantly, the increase observed in beta-cell mass was also associated with a trend towards increased beta-cell number per area (**Figure 5B**). However, beta-cell size was not changed throughout pregnancy (**Figure 5C**). We also observed a significant increase in the number of small (islet area < 1000 μm^2^) islets in the 2^nd^ (p = 0.002) and 3^rd^ trimester (p = 0.03), but a significant decrease in the number of islets with islet area between 1000-5000 μm^2^ (p = 0.01) in the 3^rd^ trimester (**Figure 5D**). Moreover, there was a trend towards increased alpha-cell mass in mid to late pregnancy, especially in the 2^nd^ trimester (p = 0.06) (**Figure 5E**). Mean islet size was significantly larger in pregnant mice during the 2^nd^ and 3^rd^ trimesters (2^nd^ trimester, p = 0.0001; 3^rd^ trimester, p = 2.2E-05) (**Figures 5F, G**).

**Figure 6:**
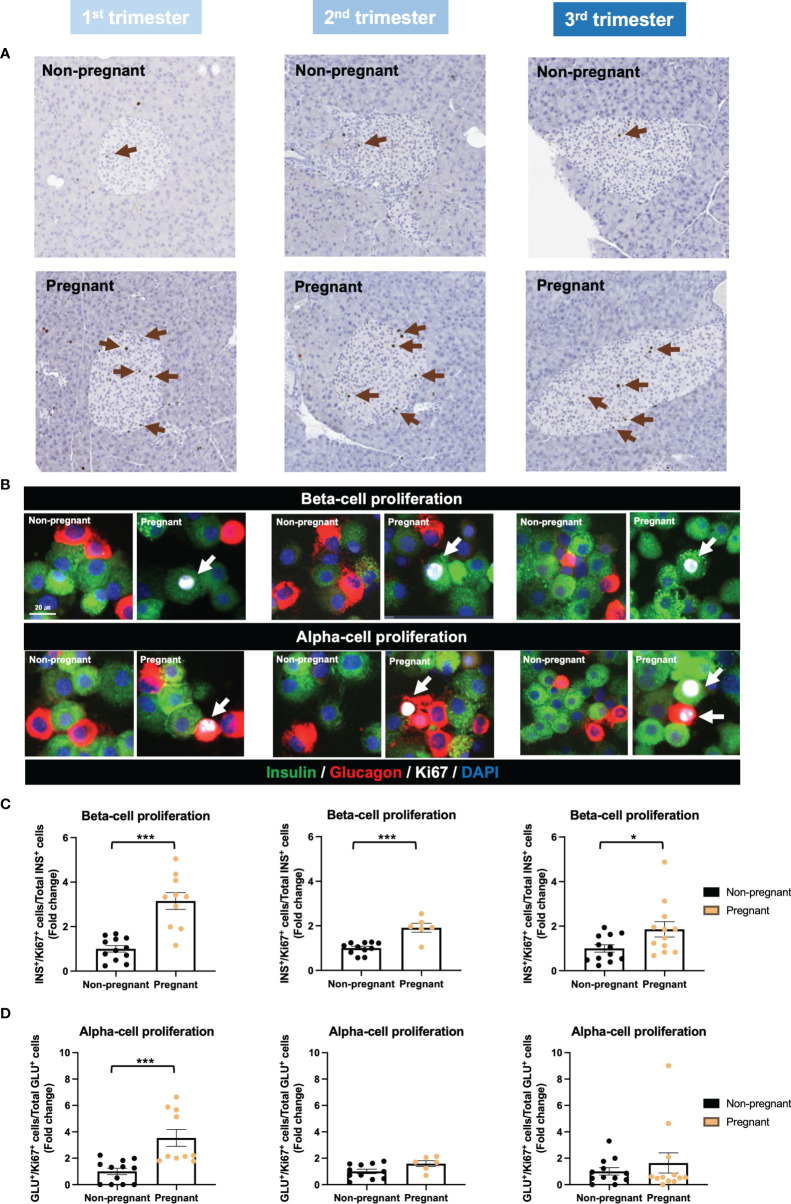
Islet proliferation in pregnant and non-pregnant mice during the three trimesters of pregnancy. **(A)** Representative images of Ki67-stained pancreatic sections from pregnant and non-pregnant mice in each trimester. **(B)** Insulin, glucagon and Ki67 staining of isolated islets using cytospin-immunofluorescent method. **(C)** Beta-cell proliferation rate. **(D)** Alpha-cell proliferation rate. Cell proliferation rate was calculated by normalizing Ki67^+^ insulin^+^/glucagon^+^ cells to total insulin^+^/glucagon^+^ cells. *P < 0.05, ***P < 0.001 compared to age-matched, non-pregnant FVB female mice.

**Figure 7:**
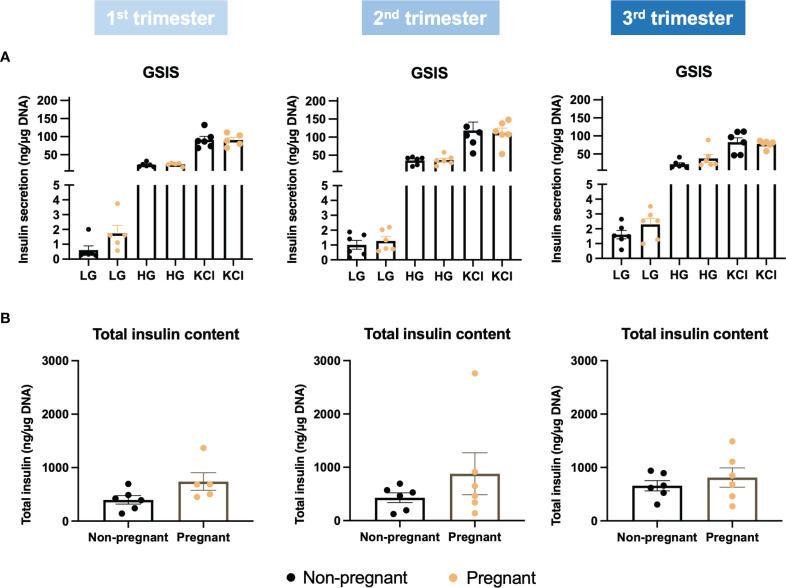
Glucose stimulated insulin secretion in islets collected during the three trimesters of pregnancy. **(A)** Insulin secretion under low glucose (2mM), high glucose (11mM) and KCl treatment. **(B)** Total insulin content. Insulin secretion and total insulin content were normalized to total DNA content/cell number.

By applying Ki67 staining, a biomarker of cell proliferation, we observed an increase of islet cell proliferation *in situ* throughout the pregnancy, compared to age-matched controls (**Figure 6A**). Using cytospin and immunofluorescence staining, we also showed a significant increase in both beta-cell and alpha-cell proliferation in the 1^st^ trimester (**Figures 6B–D**). Furthermore, beta-cell proliferation continued to increase significantly in pregnant mice during the 2^nd^ and 3^rd^ trimesters, compared to non-pregnant mice (**Figures 6B, C**). We then evaluated islet function and insulin secretion *in vitro*. In the 2^nd^ trimester, insulin secretion was increased significantly under the treatment of KCl in the pregnant mice, compared to controls (p=0.003, **Supplementary Figure 3A**). In the 3^rd^ trimester, 2mM glucose (p=0.009), 11mM glucose (p=0.049) and KCl (p=0.016) induced increased insulin secretion in the pregnant mice (**Supplementary Figure 3A**). No significant changes were observed in the total insulin content of islets derived from pregnant mice compared to controls (**Supplementary Figure 3B**). However, after normalizing the insulin secretion with total DNA content/cell number, there was no significant difference in glucose-stimulated insulin secretion or total insulin content throughout the pregnancy (**Figure 7**).

### Dynamic Changes of Metabolites in Islets During the Normal Pregnancy

**Figure 8:**
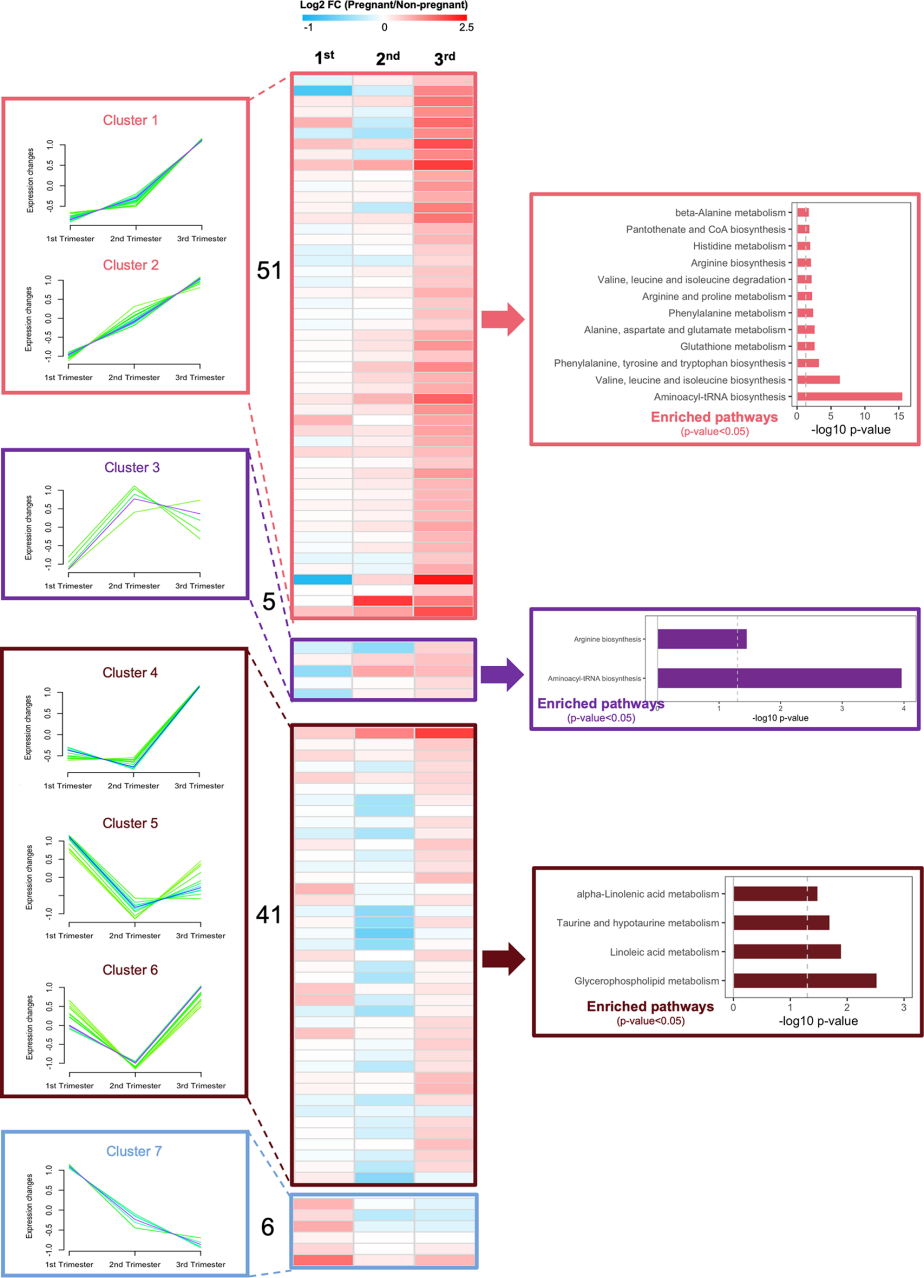
Dynamic changes of metabolic profiles in isolated islets during the three trimesters of pregnancy. Fuzzy c-means soft clustering was applied to identify the clusters of metabolites with the same trends during pregnancy. KEGG pathway analysis was performed to identify the major regulating signaling pathways within the clusters with the same trend. Fold changes are log transformed and indicated by color scale in the matrix. In heatmaps, red color indicates up-regulated in pregnant mice, whereas blue represents down-regulated in pregnant mice, compared to non-pregnant and age-matched mice.

**Figure 9:**
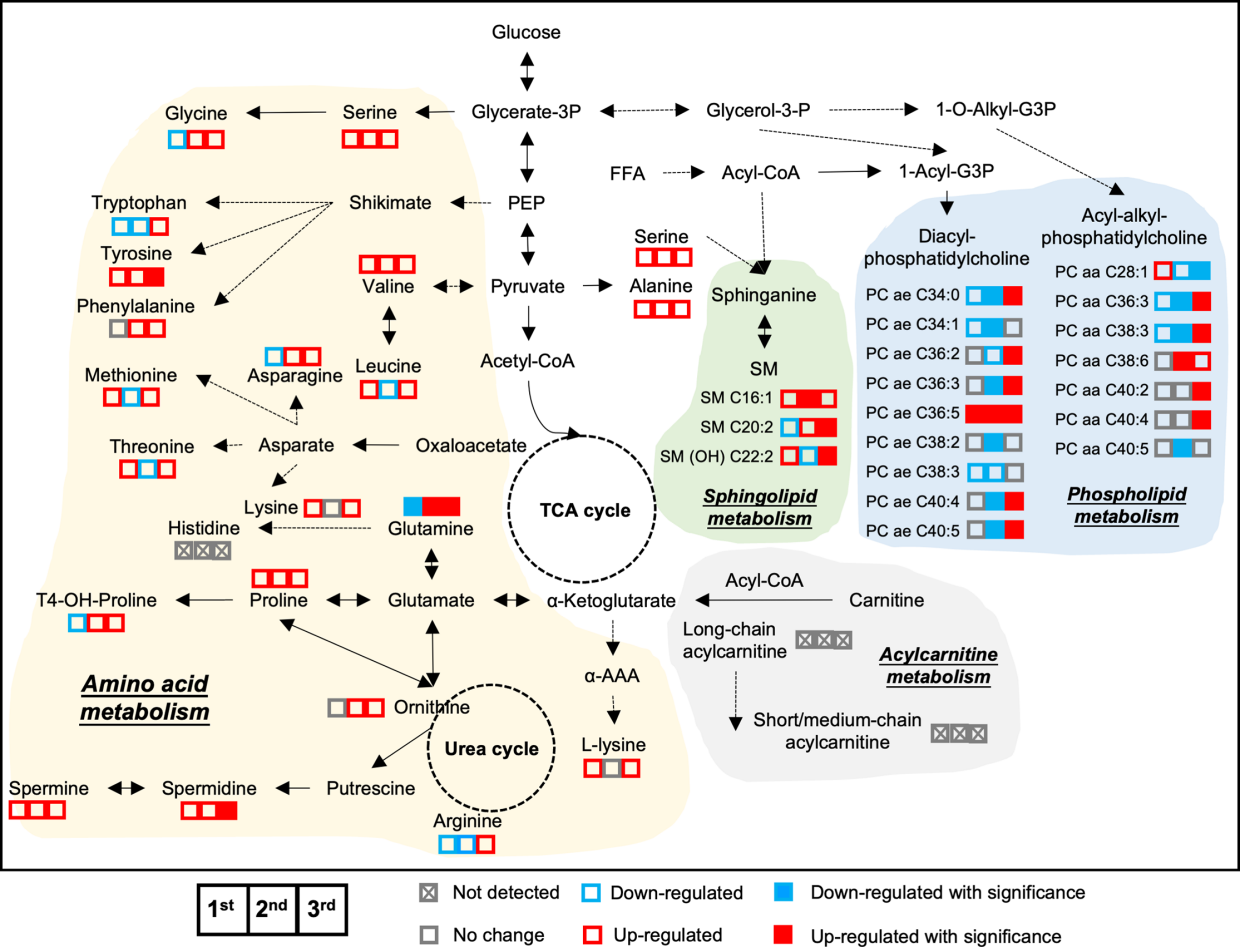
Metabolic adaptive changes in islets during pregnancy. The integrated metabolic networks of amino acid, acylcarnitine, and lipid metabolism in the islets during the three trimesters of pregnancy. Triplets of squares denote 1^st^ trimester, 2^nd^ trimester and 3^rd^ trimester data, respectively. Solid squares in red and blue represent significant changes. Empty squares denote non-significance. Red color indicates up-regulated in pregnant mice, whereas blue represents down-regulated in pregnant mice, compared to age-matched, non-pregnant female mice. Fold change value between 0.95 and 1.05 was considered no change and shown as grey color.

To better understand islet metabolism changes during normal pregnancy, we employed targeted metabolomics on islets from pregnant mice and controls at each of the three trimester time points. We identified a total of 103 metabolites in islet samples, encompassing 19 AAs, 5 BAs, 66 glycerophospholipids, and 13 SMs. By comparing pregnant mice to non-pregnant mice, the log2 fold change value of each metabolite was calculated and shown in the heatmaps (**Supplementary Figure 4**). Seven major clusters of metabolites, as well as their top corresponding significant pathways (p-value < 0.05) were identified. Notably, and in contrast to the circulating metabolome, amino acid metabolism pathways in general were upregulated throughout all three phases of pregnancy, including BCAA biosynthesis, phenylalanine, tyrosine and tryptophan biosynthesis, and glutathione metabolism (**Figure 8** and **Supplementary Figure 4**, clusters 1-3). In addition, glycerophospholid and fatty acid metabolism pathways (linoleic acid metabolism and alpha-linolenic acid metabolism) were downregulated during the early- to mid-pregnancy but reversed close to delivery (**Figure 8** and **Supplementary Figure 4**, clusters 4-6). No significantly enriched pathway was identified for cluster 7. After assimilating metabolite changes throughout the three phases of pregnancy, we observed more active AA metabolism and lipid metabolism in islets, especially during the late gestation (**Figure 9**).

Furthermore, differentially expressed metabolites were identified by comparing the pregnant mice with non-pregnant mice in each trimester (**Supplementary Figure 5**). There were no significant changes in metabolome in the islets during the early pregnancy (1^st^ trimester). In the 2^nd^ trimester, 12 phospholipids and 1 biogenic amine were changed significantly, with most of them being downregulated in pregnant mice (**Supplementary Figure 5**). In the 3^rd^ trimester, we identified 2 sphingomyelins, 20 phospholipids, 2 biogenic amines, and 3 amino acids as significantly differentially expressed between pregnant and non-pregnant mice. Most lipids and amino acids were upregulated in the late pregnancy (**Supplementary Figure 5**). Overall, the metabolome remained quite stable in the early trimester within islets but changed significantly during mid to late gestation.

## Discussion

In the present study, we have established a timed-pregnancy mouse model and applied targeted metabolomics on plasma and islet samples to evaluate adaptive metabolic changes of the whole body and islets which occur during normal pregnancy. Fasting glucose, fasting insulin, and fed glucose were lower in pregnant mice. Following the OGTT, although basal insulin was lower, insulin responses were significantly enhanced post-glucose load throughout the pregnancy. We also observed enhanced islet proliferation and progressive increases in mean islet size during pregnancy. When comparing the plasma/circulating metabolomics of pregnant and non-pregnant mice, phospholipid and fatty acid metabolism pathways were found to be upregulated throughout pregnancy, whereas amino acid metabolism pathways decreased from early- to mid-pregnancy but increased in late-pregnancy. Conversely, in islets, metabolomics revealed a consistent enrichment in amino acid metabolism pathways throughout pregnancy, with glycerophospholid and fatty acid metabolism pathways upregulated in late pregnancy.

We observed that fasting glucose, fasting insulin, and fed glucose levels were lower in pregnant mice. However, fed insulin levels were higher in pregnant mice. In human studies, fasting glucose decreases progressively with advancing gestation (1). This could be attributed to increased maternal blood volume during pregnancy (34), but also due to the insulin independent redirection of glucose disposal towards the uterus (35, 36), leading to deceased glucose levels compared to respective controls. The decreased fasting insulin levels that were observed in the present study could also be a compensatory response to the decreased fasting glucose levels. However, fed insulin levels were higher in pregnant mice compared to non-pregnant mice, which could be due to enhanced insulin secretion upon nutritional load to maintain glucose homeostasis during pregnancy.

We performed OGTTs to evaluate the glucose homeostasis in pregnant and non-pregnant mice throughout the pregnancy. We found that the AUC of glucose during the OGTT was decreased in the pregnant mice, especially in the mid- to late-pregnancy, and that the insulin response during the first 10 minutes of OGTT was higher in the pregnant mice. During pregnancy, islets undergo compensatory morphological changes such as increases in beta-cell mass, which are likely achieved through a combination of hypertrophic expansion, islet proliferation, potential neogenesis from precursor cells as well as a decrease in apoptosis (34, 37, 38). In addition, there are functional changes in islets, including increased insulin production and secretion (39, 40).

In the present study, both alpha and beta cell proliferation were found to be significantly elevated in early to mid-pregnancy, a probable cause of the increased islet size seen in mid to late gestation. It has been reported that the functional and morphological adaptions in the islets occur in response to pregnancy itself. Szlapinski et al. showed a significantly increased alpha-cell proliferation at GD 9.5 in mouse pregnancies, resulting in an increased alpha-cell mass at GD 18.5 (41). Beta-cell proliferation and beta-cell mass were also observed to increase dramatically during the pregnancy (39). The proliferation of islet cells may be attributed to multiple pregnancy hormones, including prolactin (PRL) and placental lactogen (PL), which are required for increasing beta-cell mass during pregnancy (42–44). Multiple genes have been noted to be involved in this process, which are primarily downstream of lactogens (39, 45, 46). However, we showed that glucose-stimulated insulin secretion was not different between the pregnant and control islets at any stage of pregnancy when normalized to DNA content, suggesting the increased insulin secretion that we observe was likely due to increased beta cell number/mass and thus increased secretory capacity.

Applying targeted metabolomics to fasting plasma, we noticed an up-regulation of phospholipid and fatty acid metabolism throughout the pregnancy. Lipid metabolism during pregnancy has been divided into 2 phases: anabolic and catabolic (4, 47). During the first 2 trimesters of human gestation, fat is stored in the fat deposits, whereas in later pregnancy (3rd trimester), fat tissue is broken down (4, 48–51). Enhanced *de novo* lipogenesis and maternal hyperphagia are factors contributing to early pregnancy anabolism; whereas in late pregnancy, enhanced adipose tissue lipolytic activity and reduced activity of adipose tissue lipoprotein lipase (LPL) lead to accelerated breakdown of fat deposits as well as decreased fat deposits. One striking example of this mobilization of lipid depots is the increase in free fatty acid and phospholipid concentrations in maternal plasma with advancing gestation (52–54), which was also shown in our study. We also observed that in circulation, AA metabolism was decreased in the early- to mid-pregnancy and increased in later pregnancy (GD19) close to delivery. It has been demonstrated that circulating concentrations of maternal amino acids are decreased during pregnancy (55–57). Specifically, most amino acid levels decrease in the early pregnancy, remain at a lower level throughout later pregnancy, and increase to non-pregnant levels after delivery (57, 58). Reduced amino acid levels in early pregnancy could in part be attributed to the changes in renal function since amino acids are filtered by the glomerulus and reabsorption of amino acids is decreased during pregnancy (58–60). Additionally, these changes in amino acid concentrations are likely affected by the increased need for protein synthesis (61).

Our islet-specific metabolomics data showed that there was an activation of amino acid metabolism pathways within the islets throughout normal pregnancy. Additionally, valine, glutamate and tyrosine were found to be significantly upregulated in pregnant mice in the 3rd trimester. In islets and beta-cell lines, specific amino acids (L-arginine, L-lysine, L-alanine, L-proline, L-leucine, L-valine, L-glutamate and L-glutamine) have been shown to be associated with enhanced insulin secretion under glucose stimulation (62–66). For example, mitochondrial glutamate is involved in glucose-induced insulin exocytosis (67), and cytosolic glutamate plays a key role in linking glucose metabolism to incretin/cAMP action to amplify insulin secretion (68). Branched-chain amino acids, consisting of valine, leucine and isoleucine are also reported to mediate insulin exocytosis (69, 70), which could be due to upregulated glucokinase, increased anaplerosis and TCA activity in the beta-cell (70, 71). These data suggested the enhanced insulin response after glucose load that was observed in pregnant mice could be attributed to the accumulation of specific amino acids within islets.

Very few studies have evaluated the effects of amino acids on the proliferation of islets. Amino acids enter the cells through transporters, such as the L-amino acid transporter 1 (LAT1), which is expressed abundantly in the islets, and knocking down LAT1 in beta-cells and islets induced an inhibition of leucine-stimulated mTORC1 activation and islet cell proliferation (72). Dean et al. and Kim et al. showed that blocking the action of glucagon on the alpha-cell resulted in elevated circulating amino acids and led to increased alpha-cell proliferation, which may be linked by amino acid transporter Slc38a5 (73, 74). In addition, calcium sensing receptor (CASR) expressed in pancreatic islets, which has affinity for several amino acids, was shown to be associated with increased islet function and alpha-cell proliferation, suggesting that the CASR pathway plays a key role in regulating islet function and mass (75). Similarly, GPR142 agonists and its endogenous ligands tryptophan and phenylalanine were shown to stimulate beta-cell proliferation and insulin secretion, which may act through alpha-cell derived glucagon-like peptide 1 (GLP-1) stimulation of beta-cells (76–78). On the contrary, Mullooly et al. showed that elevated levels of branched-chain amino acids have little effect on islet viability, but increased levels of L-arginine were beta-cell toxic, leading to decreased islet proliferation and increased islet cell apoptosis, through the elicitation of an endoplasmic reticulum stress response (79). Based on these results, further studies focused on the effect of amino acids on islet proliferation are required.

There was a fluctuation in lipid metabolism (glycerophospholipid and fatty acid metabolism) throughout pregnancy, with most phospholipids/sphingolipids being upregulated in pregnant mouse islets during late pregnancy compared to controls (**Supplementary Figure 3**). Lipids increase in the beta cell during glucose stimulation that coincides with insulin secretion (80). This supports lipids and lipid remodeling as necessary during insulin biosynthesis and exocytosis. The phospholipids within insulin secretory granules (ISG) are shown to be in a dynamic state and facilitate fusion of ISG with the plasma membrane, enhancing the glucose-stimulated insulin exocytosis (81). However, the proposed effects of lipids on islet proliferation and function currently diverge greatly. Several preclinical studies showed that fatty acids along with glucose lead to insulin resistance and a marked increase in the beta-cell proliferation and islet size, but these effects were not observed with the infusion of fatty acids alone (82–85). In marked contrast, Pascoe et al. found that fatty acid infusion in mice blocked glucose-induced beta-cell proliferation *in vivo* (86). The effects of fatty acids on islets proliferation also depends on the degree of unsaturation. Saturated fatty acids such as palmitate are associated with beta-cell dysfunction, whereas monounsaturated fatty acids such as oleate promote beta-cell proliferation and protect beta-cells against the toxic effects of palmitate (87, 88). It is noteworthy that besides metabolites and pathways examined in the present study, the proliferation of islets may also be associated with other pathways/genetic targets that we were unable to evaluate. During pregnancy, several genes have been shown to be upregulated that are likely influencing the degree of proliferation during pregnancy (89). As such, investigating the effects of metabolites on proliferation and changes related to pregnancy, such as modifications at the gene level should be further investigated.

There are of course some limitations to this study. First, there are biological and physiological differences between humans and mice, which should be considered particularly when translating research from rodents to human populations. Second, the number of animals used in this study is limited. Despite these limitations, this study provides insight into the metabolic adaptions during pregnancy, providing a molecular rationale to further explore the regulation of maternal metabolism and pregnancy disorders, like gestational diabetes.

## Data Availability Statement

The original contributions presented in the study are included in the article/**Supplementary Material**. Further inquiries can be directed to the corresponding authors.

## Ethics Statement

The animal study was approved by University of Toronto animal care committee (#20011576).

## Author Contributions

ZZ: Conceptualization, data analysis, investigation, visualization, methodology, writing-original draft preparation, writing-review and editing. AP: Conceptualization, writing-original draft preparation, writing-review and editing. FD: Conceptualization, supervision, investigation, writing-original draft preparation and editing. MW: Conceptualization, supervision, resources, funding acquisition, methodology, investigation, writing-original draft preparation, writing-review and editing. All authors read and approved the final manuscript.

## Funding

This study is supported by research grants from Canadian Institutes of Health Research (CIHR): FRN 143219 (MW), https://www.cihr-irsc.gc.ca. National Natural Science Foundation of China: 82100702 (ZZ), https://www.nsfc.gov.cn. China Scholarship Council fellowships (ZZ), https://www.csc.edu.cn/. Banting & Best Diabetes Centre (ZZ and AP) https://bbdc.org/. The funders had no role in study design, data collection and analysis, decision to publish, or preparation of the manuscript.

## Conflict of Interest

The authors declare that the research was conducted in the absence of any commercial or financial relationships that could be construed as a potential conflict of interest.

## Publisher’s Note

All claims expressed in this article are solely those of the authors and do not necessarily represent those of their affiliated organizations, or those of the publisher, the editors and the reviewers. Any product that may be evaluated in this article, or claim that may be made by its manufacturer, is not guaranteed or endorsed by the publisher.
