# Supplementary material for: Adaptive Changes in Glucose Homeostasis and Islet Function During Pregnancy: A Targeted Metabolomics Study in Mice
Source: Front Endocrinol (Lausanne). 2022 May 4;13:852149. doi: 10.3389/fendo.2022.852149 (PMC9116578; doi:10.3389/fendo.2022.852149)
Supplement: Supplementary file 1 [file DataSheet_1.pdf]

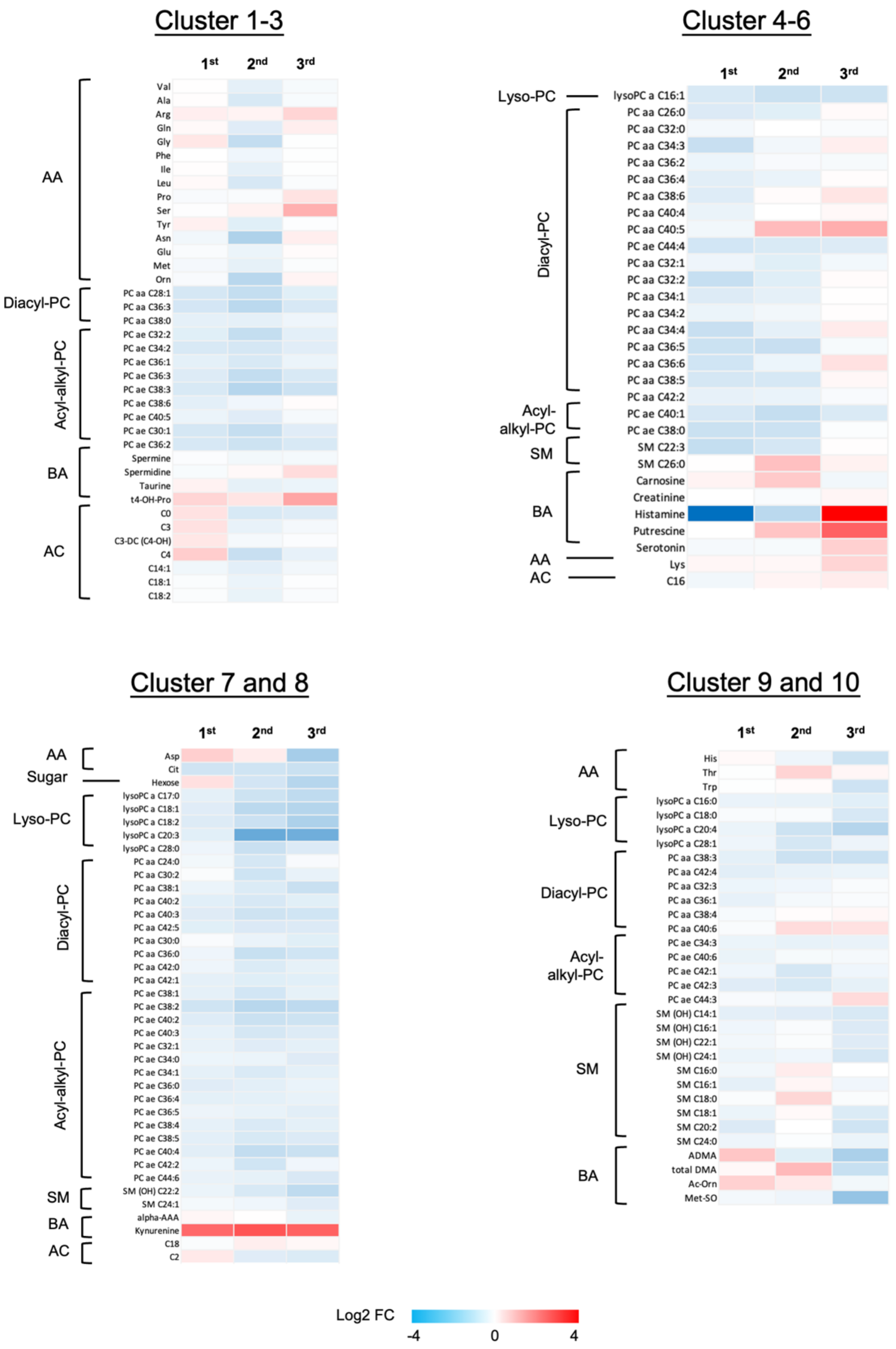

Supplementary Figure 1. Dynamic changes of metabolic profiles in the fasting plasma during the three trimesters of pregnancy. Fold changes are log transformed and indicated by color scale in the matrix. Metabolites within each cluster were indicated. Red color indicates up-regulated in pregnant mice, whereas blue represents down-regulated in pregnant mice, compared to age-matched, non-pregnant female mice.

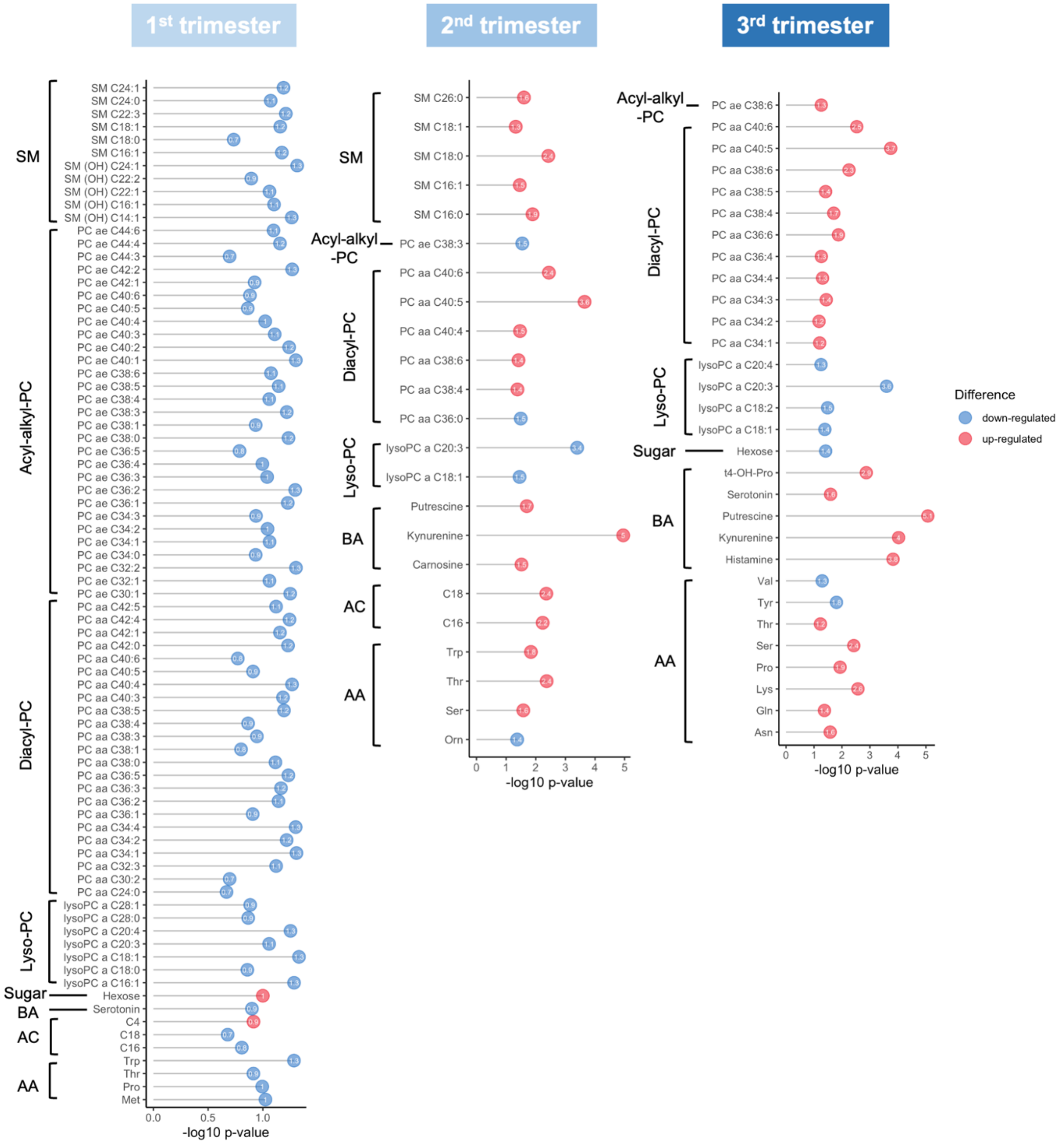

Supplementary Figure 2. Differentially expressed metabolites in circulation during the three trimesters of pregnancy. Bubble plots showing the differentially expressed metabolites (FDR<0.3) with the –log10 p-values. Red indicates up-regulated in pregnant mice, blue indicates down-regulated in pregnant mice, compared to age-matched, non-pregnant female mice.

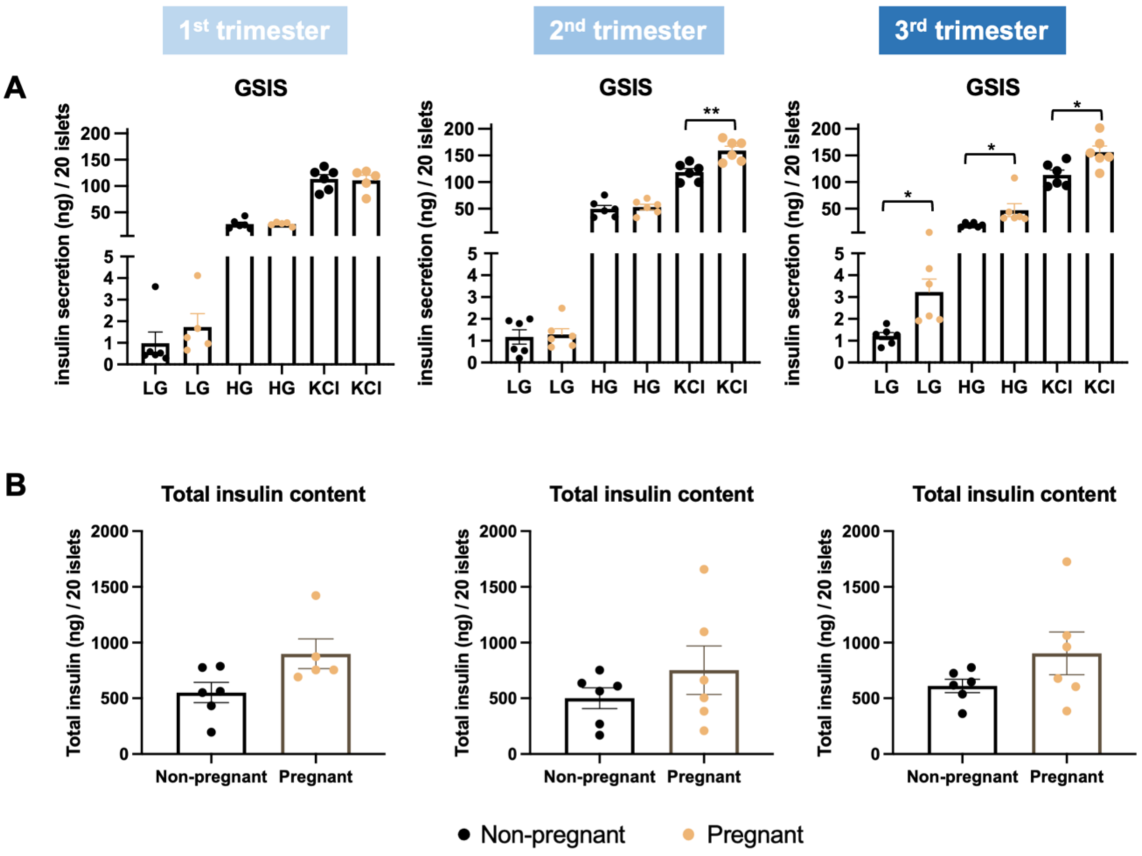

Supplementary Figure 3. Glucose stimulated insulin secretion in islets collected during the three trimesters of pregnancy. A) Insulin secretion of 20 isolated islets under low glucose (2mM), high glucose (11mM) and KCl treatment. B) Total insulin content of 20 isolated islets. *P<0.05, **P<0.01, ***P<0.001, compared to age-matched, non-pregnant FVB female mice.

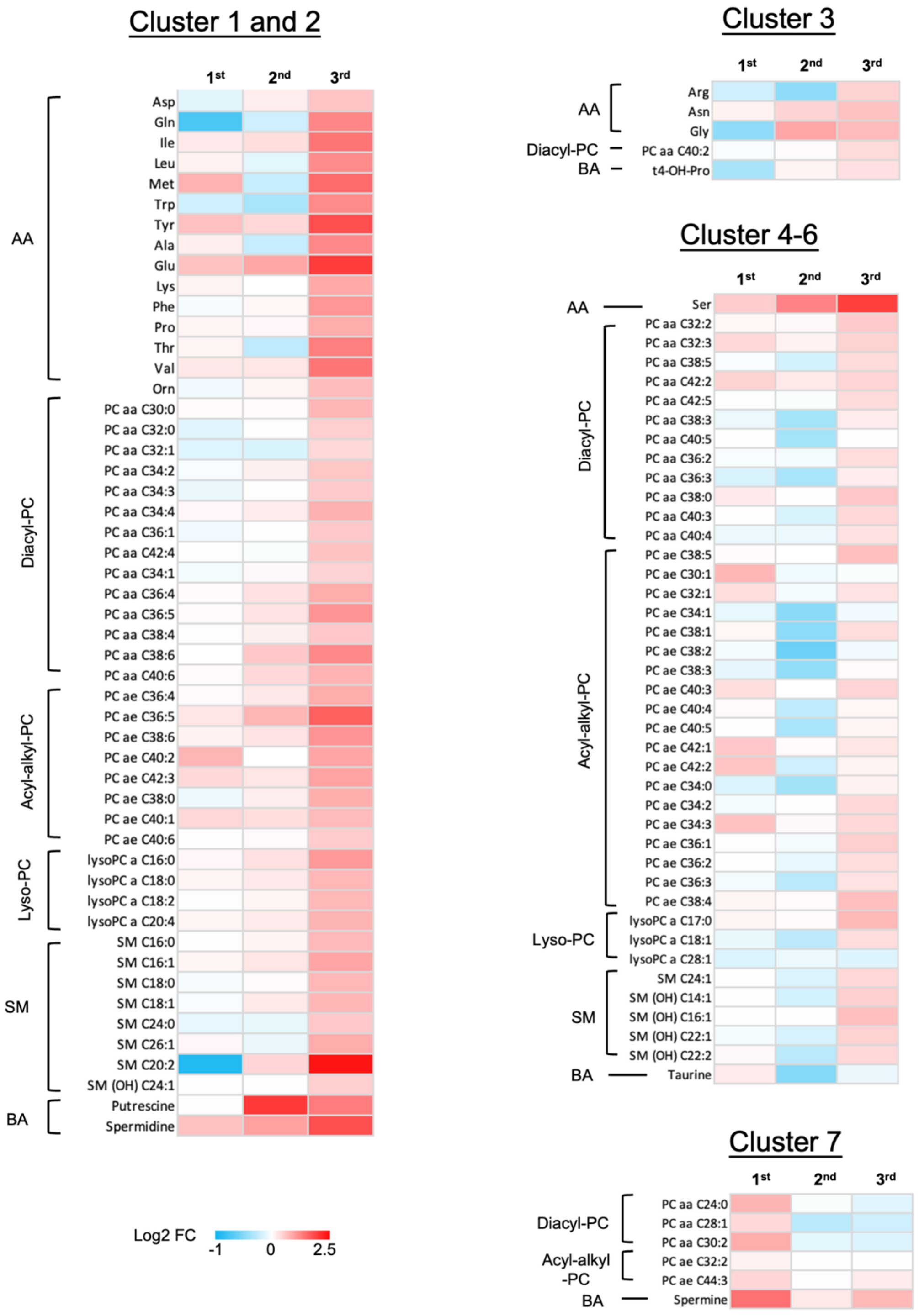

Supplementary Figure 4. Dynamic changes of metabolic profiles in isolated islets during the three trimesters of pregnancy. Fold changes are log transformed and indicated by color scale in the matrix. Metabolites within each cluster were indicated. Red color indicates up-regulated in pregnant mice, whereas blue represents down-regulated in pregnant mice, compared to age-matched, non-pregnant female mice.

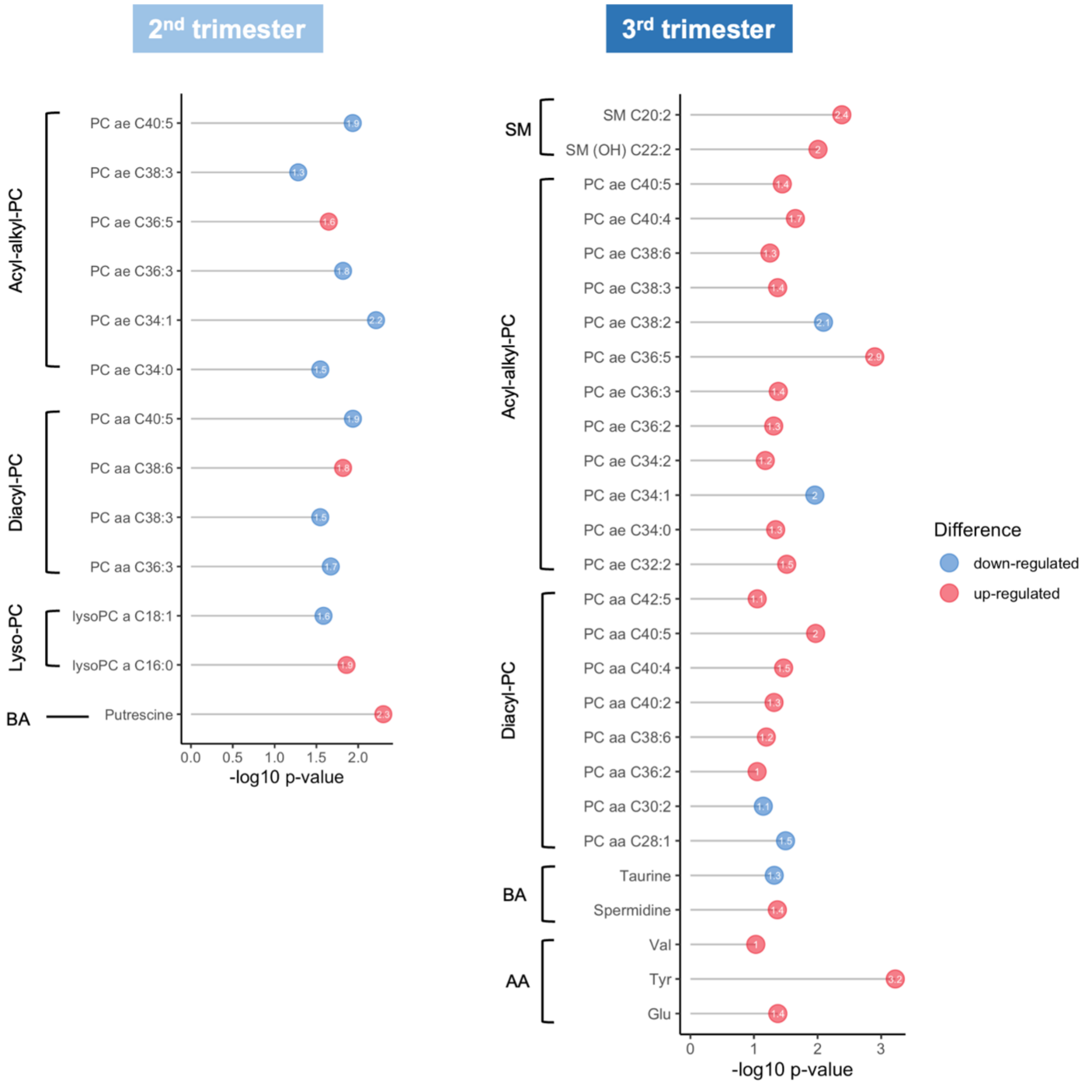

Supplementary Figure 5. Differentially expressed metabolites in isolated islets during the three trimesters of pregnancy. Bubble plots showing the differentially expressed metabolites (FDR<0.3) with the –log10 p-values. Red indicates up-regulated in pregnant mice, blue indicates down-regulated in pregnant mice, compared to age-matched, non-pregnant female mice.
